# Lysine-222 succinylation reduces lysosomal degradation of lactate dehydrogenase a and is increased in gastric cancer

**DOI:** 10.1186/s13046-020-01681-0

**Published:** 2020-08-28

**Authors:** Xiang Li, Chen Zhang, Ting Zhao, Zhongping Su, Mengjing Li, Jiancheng Hu, Jianfei Wen, Jiajia Shen, Chao Wang, Jinshun Pan, Xianmin Mu, Tao Ling, Yingchang Li, Hao Wen, Xiaoren Zhang, Qiang You

**Affiliations:** 1 Affiliated Cancer Hospital & Institute of Guangzhou Medical University, Guangzhou, 510095 China; 2 Department of Biotherapy, Department of Surgery, Second Affiliated Hospital of Nanjing Medical University, Nanjing, 210011 China; 3 Nanjing University of Chinese Medicine, Nanjing, 210023 China; 4 Division of Cellular and Molecular Research, National Cancer Centre Singapore, Singapore, 169610 Singapore; 5 Cancer and Stem Cell Biology Program, Duke-NUS Medical School, Singapore, 169857 Singapore; 6 The First Affiliated Hospital of Nanjing Medical University, Nanjing, 210029 China; 7 Key Laboratory of Cell Homeostasis and Cancer Research of Guangdong Higher Education Institutes, Guangzhou Medical University, Guangzhou, 510182 China

**Keywords:** Lysine succinylation, Gastric cancer, LDHA, SQSTM1, CPT1A

## Abstract

**Background:**

Lysine succinylation is an emerging posttranslational modification that has garnered increased attention recently, but its role in gastric cancer (GC) remains underexplored.

**Methods:**

Proteomic quantification of lysine succinylation was performed in human GC tissues and adjacent normal tissues by mass spectrometry. The mRNA and protein levels of lactate dehydrogenase A (LDHA) in GC and adjacent normal tissues were analyzed by qRT-PCR and western blot, respectively. The expression of K222-succinylated LDHA was measured in GC tissue microarray by the K222 succinylation-specific antibody. The interaction between LDHA and sequestosome 1 (SQSTM1) was measured by co-immunoprecipitation (co-IP) and proximity ligation assay (PLA). The binding of carnitine palmitoyltransferase 1A (CPT1A) to LDHA was determined by co-IP. The effect of K222-succinylated LDHA on tumor growth and metastasis was evaluated by in vitro and in vivo experiments.

**Results:**

Altogether, 503 lysine succinylation sites in 303 proteins were identified. Lactate dehydrogenase A (LDHA), the key enzyme in Warburg effect, was found highly succinylated at K222 in GC. Intriguingly, this modification did not affect LDHA ubiquitination, but reduced the binding of ubiquitinated LDHA to SQSTM1, thereby decreasing its lysosomal degradation. We demonstrated that CPT1A functions as a lysine succinyltransferase that interacts with and succinylates LDHA. Moreover, high K222-succinylation of LDHA was associated with poor prognosis in patients with GC. Finally, overexpression of a succinylation-mimic mutant of LDHA promoted cell proliferation, invasion, and migration.

**Conclusions:**

Our data revealed a novel lysosomal pathway of LDHA degradation, which is mediated by the binding of K63-ubiquitinated LDHA to SQSTM1. Strikingly, CPT1A succinylates LDHA on K222, which thereby reduces the binding and inhibits the degradation of LDHA, as well as promotes GC invasion and proliferation. This study thus uncovers a new role of lysine succinylation and the mechanism underlying LDHA upregulation in GC.

## Background

Protein posttranslational modification (PTM) is a dynamic, reversible modification process, and is one of the two main mechanisms that enable the expansion of proteomic diversity [1]. Hundreds of PTMs have been discovered, including phosphorylation, methylation, acetylation, glycosylation, succinylation, biotinylation, ubiquitination, SUMOylation, propionylation, butyrylation, lactylation, etc. [2–4]. Succinylation of lysine is an evolutionarily conserved PTM whereby succinyl groups are transferred from succinyl-CoA to the specific alpha-amino of lysine [5]. In 2011, succinylation was first shown as a naturally occurring PTM on lysine residues in bacteria [6]. Since then, numerous studies discovered proteins that are targeted by succinylation [7]. For example, the succinylation of uncoupling protein 1 (UCP1), a key enzyme in brown adipose tissue thermogenesis, leads to a decrease in its stability and activity, while the increase in the succinylation of UCP1 leads to impaired mitochondrial enzyme activity and respiratory function [8]. Under nutrient deficiency, succinylated pyruvate kinase M2 (PKM2) can migrate into mitochondria and bind to voltage-dependent anion channel 3 (VDAC3) to improve mitochondrial permeability and thereby generate more ATP to promote cell survival [9]. Strikingly, lysine acetyltransferase 2A (KAT2A) and carnitine palmitoyltransferase 1A (CPT1A) were recently uncovered as succinylating enzymes [10–12]. However, the role of lysine succinylation in GC remains poorly understood. Our previous results showed that succinylation was more a consistent protein modification than crotonylation and acetylation in GC and adjacent tissues, and succinylation of S100A10 by CPT1A promoted human gastric cancer (GC) invasion [13].

Changes in energy metabolism are one of the characteristics of tumor cells. Malignant cells are inclined to obtain energy for rapid growth and reproduction through glycolysis even under aerobic conditions. This phenomenon is called aerobic glycolysis, or Warburg effect [14, 15]. Lactate dehydrogenase A (LDHA) is a key enzyme in aerobic glycolysis, and is mainly expressed in skeletal muscle, where it preferentially converts pyruvate to lactic acid and NADH to NAD^+^ [16, 17]. LDHA is also highly expressed in a variety of tumors, including GC, esophageal cancer, pancreatic cancer, cholangiocarcinoma, breast cancer, cervical cancer, renal cell cancer, lymphoma, and neuroblastoma [18–22]. High expression of LDHA is often associated with poor prognosis and high metastasis rate [19]. Therefore, LDHA is considered a promising new target in the prevention and treatment of tumors [23, 24].

In this study, we aimed to explore the role of lysine succinylation in GC and identify succinylated targets that could be associated with cancer progression and prognosis. To this aim, we performed quantitative lysine succinylome analysis in human GC tissues and adjacent normal tissues using TMT labeling and affinity enrichment followed by high-resolution LC-MS/MS analysis. Moreover, we investigated the mechanism underlying high expression of LDHA in GC.

## Methods

### GC samples

The tumor samples in this study were taken from seven patients with primary gastric adenocarcinoma who had not received chemotherapy or any other treatment before surgery and were surgically removed with the patient’s informed consent. The patients were from the Second Affiliated Hospital of Nanjing Medical University (Jiangsu, China). This study was approved by the Hospital Ethics Committee of the Second Affiliated Hospital of Nanjing Medical University and carried out in accordance with the principles of the Declaration of Helsinki.

### Proteomic quantification of lysine Succinylation

The corresponding tumor and adjacent normal tissues obtained from the patients were used to quantify dynamic changes of lysine succinylome in PTM Biolabs (Hangzhou, China). The experiment included TMT labeling, HPLC fractionation, affinity enrichment, and mass spectrometry-based quantitative proteomics. Intensive bioinformatic analysis was then carried out to annotate the quantifiable targets, including protein annotation, functional classification, functional enrichment, functional enrichment-based cluster analysis, etc.

### RNA isolation and qPCR

Total RNA was extracted using TRIzol reagent (Invitrogen, Carlsbad, CA, USA) and transcribed into cDNA using the reverse transcription kit (Takara Biotechnology, Beijing, China) following the manufacturers’ instructions. RNA was analyzed using real-time qPCR with SYBR Green PCR Master mix (Roche Applied Science, Mannheim, Germany) on a StepOnePlus™ real-time PCR System (Applied Biosystems, Foster City, CA, USA). The relative gene expression was normalized to GAPDH. Specific primer sets used for this assay included LDHA (forward: GGA TCT CCA ACA TGG CAG CCT T, reverse: AGA CGG CTT TCT CCC TCT TGC T), and GAPDH (forward: TTG CCA TCA ATG ACC CCT TCA, reverse: CGC CCC ACT TGA TTT TGG A).

### Immunohistochemical analysis

The tissues were fixed in 10% formalin, and then embedded in paraffin. The specific steps were performed as previously described [13]. Rabbit polyclonal antibodies against the human LDHA K222-succinylated peptide, PDLGTDK (suc) DKEQWK, were generated from two rabbits (#1 and #2) at ChinaPeptides Co., Ltd. (Shanghai, China). For immunohistochemical staining, 5-μm-thick serial sections were used to prepare the slides. Antigen retrieval was performed with 10 mM citrate antigen retrieval solution (CW Biotech, Beijing, China). Anti-LDHA (#3582S, Cell Signaling Technology, Danvers, MA, USA) and anti-LDHA-K222suc antibodies was used at a dilution of 1:400. The immunostaining index was based on the staining intensity and percentages of positively stained tumor cells. The intensity was scored between 0 and 3: 0 (no staining), 1 (weak staining), 2 (moderate staining), and 3 (strong staining). The percentages of positively stained tumor cells were defined as 0 (< 10%), 1 (10 –30%), 2 (31 –50%), and 3(> 50% of positive cells) [25]. The staining index was then calculated as the score of staining intensity multiplied by the percentage of positively stained tumor cells. Tumor stage was classified according to the seventh edition of the UICC/AJCC TNM staging system.

### Cell culture and treatment

AGS, HGC27, B16-F10, and 293 T cells were purchased from Shanghai Cafa Biological Technology Co. Ltd. (Shanghai, China), tested negative for mycoplasma, and authenticated by Genetic Testing Biotechnology Corporation (Suzhou, China) using short tandem repeat markers. The cells were cultured in Dulbecco’s Modified Eagle Medium (DMEM; Invitrogen, Carlsbad, CA, USA) supplemented with 10% fetal bovine serum (FBS; Gibco, Grand Island, NY, USA) and 1% penicillin/streptomycin (Thermo Scientific, Waltham, MA, USA), and maintained in a humidified incubator at 37 °C with 5% CO_2_. The following reagents were used to treat the cells: MG132 (10 mM, #S2619, Selleck, Shanghai, China), cycloheximide (CHX, 10 μg/ml, #HY-12320, Selleck, Shanghai, China), Bafilomycin A1 (Baf-A1, 20 nM, #S1413, Selleck, Shanghai, China) and chloroquine (50 μM, #C6628, Sigma, St. Louis, MO, USA).

### Generation of stable cell lines

The stable cell lines were generated using lentivirus system. Briefly, the genes were cloned into the specific vector pLJM1, and then transfected into HEK293T cells using Lipofectamine 3000 reagent. Lentiviral supernatants were harvested from HEK293T cells and mixed together with 8 μg/mL of polybrene to increase the infection efficiency. The infected cancer cells were then selected in culture media containing 2 μg/ml of puromycin for 2 weeks.

### Immunofluorescence staining for colocalization study

AGS cells (5 × 10^5^) were seeded and cultured in 12-well plates in the presence or absence of BafA1 for 24 h. Then, the cells were fixed with 4% paraformaldehyde for 15 min, washed three times with PBS for 5 min, and permeabilized with 0.1% Triton x-100 for 10 min. After blocking for 1 h in 3% bovine serum albumin (BSA) at 37 °C, the cells were incubated overnight at 4 °C with primary antibodies anti-LDHA (#3582S, Cell Signaling Technology, Danvers, MA, USA) and anti-SQSTM1/p62 (#88588S, Cell Signaling Technology). Subsequently, the secondary antibodies were added for 1 h at 37 °C followed by counterstaining with DAPI. The cells were then observed and photographed under the confocal microscope (Olympus FV-1000).

### Plasmid construction and cell transfection

Full-length WT cDNA or cDNA with point mutations of the *LDHA* gene was synthesized (Wuxi Qinglan Biotech. Inc., Yixing, China) and cloned into indicated vectors including pRF-FLAG or pRF-HA (kindly obtained from Prof. Hongbing Shu). CPT1A and KAT2A cDNA plasmids were purchased from Sino Biological (Beijing, China) and subsequently cloned into the pRF-FLAG vector. HA tagged ubiquitin (Ub), HA tagged ubiquitin with only K48 (K48) and HA tagged ubiquitin with only K63 (K63) were kindly obtained from Prof. Yongzhong Liu. Lipofectamine 3000 (Invitrogen) was used for cell transfection followed by the manual.

### RNA interference analysis

*CPT1A* shRNA and control shRNA plasmids were purchased from Shanghai Genechem Co., Ltd. (Shanghai, China) and used as before [13]. The shRNA sequences for *CPT1A* are as follows: (#1) TAG CCT TTG GTA AAG GAA T, (#2) ATG TTA CGA CAG GTG GTT T, (#3) CAA CGA TGT ACG CCA AGA T. The transfections were performed with Lipofectamine 3000. The protein samples were collected for WB detection after transfection for 24 h.

### Immunoprecipitation

The co-immunoprecipitation (co-IP) assay was performed as described before [13]. In brief, cells were lysed in co-IP buffer (20 mM Tris, pH 7.5, 150 mM NaCl, 1% Triton X-100, and 1 mM EDTA) containing protease inhibitors (Roche Applied Science, Mannheim, Germany) on ice for 30 min. Then, the cells were centrifuged, and the supernatant was collected, followed by incubation with primary antibodies and GammaBind Plus Sepharose (#17088601; GE Healthcare, Logan, UT, USA) with gentle rocking overnight at 4 °C. The next day, the mixture was pelleted, washed six times with cold 1× co-IP buffer, and then analyzed by western blotting.

### Proximity ligation assay (PLA) assay

The Duolink® PLA assay was performed as indicated in the manual. In brief, AGS cells were treated as indicated and stained with mouse anti-SQSTM1 and rabbit anti-LDHA antibodies as described for the immunofluorescent staining. Duolink® PLA was then performed using the anti-rabbit PLUS (#DUO92002, Sigma, St. Louis, MO, USA) and anti-mouse MINUS (#DUO92004, Sigma, St. Louis, MO, USA) probes. Following probe incubation, ligation, and amplification, the cells were observed and photographed under the confocal microscope (Olympus FV-1000).

### Western blotting

Total proteins were extracted from GC tissues or cells using RIPA lysis buffer containing protease inhibitor cocktail (Roche Applied Science, Mannheim, Germany). The lysates were mixed with SDS loading buffer, boiled for 8 min, resolved by SDS-PAGE, and then transferred to PVDF membranes (Millipore, Bedford, MA, USA). After blocking with 5% nonfat milk, the membranes were incubated with primary antibodies: anti-LDHA (#3582S, Cell Signaling Technology), anti-HA (clone 3F10, #11867423001, Roche), anti-FLAG M2 (#F1804; Sigma), anti-His TAG (#12698S, Cell Signaling Technology), pan succinyl-lysine antibody (#PTM-401; PTM Bio, Hangzhou, China), anti-β-actin (#4970; Cell Signaling Technology), or anti-CPT1A antibody (#12252; Cell Signaling Technology).

### Colony formation assay

A total of 800 AGS cells stably expressing LDHA or LDHA variants were seeded in 6-well plates, cultured for about 14 days. Then, the cells were fixed with 70% methanol and stained with Giemsa solution. Colonies containing more than 50 cells were considered as survivors.

### Cell invasion assay

The cell invasion assay was performed in a 24-well Transwell Chamber (Costar, Corning, NY, USA) coated with Matrigel (BD Pharmingen, San Jose, CA, USA). AGS cells (2 × 10^5^ /200 μl) were cultured in the upper compartment in serum-free medium. In the lower compartment, 10% complete medium was added. After incubation at 37 °C for 24 h, the cells were fixed with 4% paraformaldehyde, stained by crystal violet, and then photographed under a microscope.

### Wound-healing assay

Cells were seeded and cultured in a 6-well plate until a confluent monolayer was formed. A sterile plastic tip was used to scratch on the monolayer of cells. Pictures were taken with a microscope at the specified timepoints to observe the migration distance. Migration was quantified as a percentage of wound closure.

### Xenograft model

Male nude mice (4–6 weeks old) were purchased from Model Animal Research Center of Nanjing University (Nanjing, Jiangsu, China). All animal studies were approved by the Nanjing Medical University Ethics Review Board. Approximately 5 × 10^6^ cells stably expressing Flag-LDHA, Flag-K222R, or K222E were subcutaneously injected into the nude mice. The tumor tissues were removed after 4 weeks, and the mice were euthanized. Tumor volume was calculated as width × length × (width + length)/2. LDHA levels were examined by western blotting. The lactate levels were measured by lactic acid assay kit (Nanjing Jiancheng Bioengineering Institute, Nanjing, China) followed by the manual.

### Assessment of melanoma lung metastasis

The mouse model of melanoma lung metastasis was employed following a previously established method [13, 26]. In brief, B16-F10 cells were administered to the mice by tail-vein injection (2 × 10^5^ cells/mouse in 200 μl DMEM) (*n* = 5 in each group). Lung melanoma metastases were determined by the number of colonies that appeared as black dots on the pleural surface.

### Statistical analysis

Statistical analysis was performed using GraphPad Prism software (version 7.0; La Jolla, CA). All values are expressed as mean ± standard error of the mean. Differences between groups were assessed by means of a two-tailed unpaired Student t test or ANOVA for comparison of two or multiple groups, respectively. When ANOVA was significant, post hoc testing of differences between groups was performed using the Least Significant Difference (LSD) test. The categorical variables were compared using the Chi-squared test. Overall survival was calculated using the Kaplan-Meier method and the Gehan-Breslow-Wilcoxon test. All experiments were repeated at least 3 times, and *P* < 0.05 was considered significant.

## Results

### The protein succinylation landscape significantly differs between gastric cancer tissues and adjacent normal tissues

**Fig. 1:**
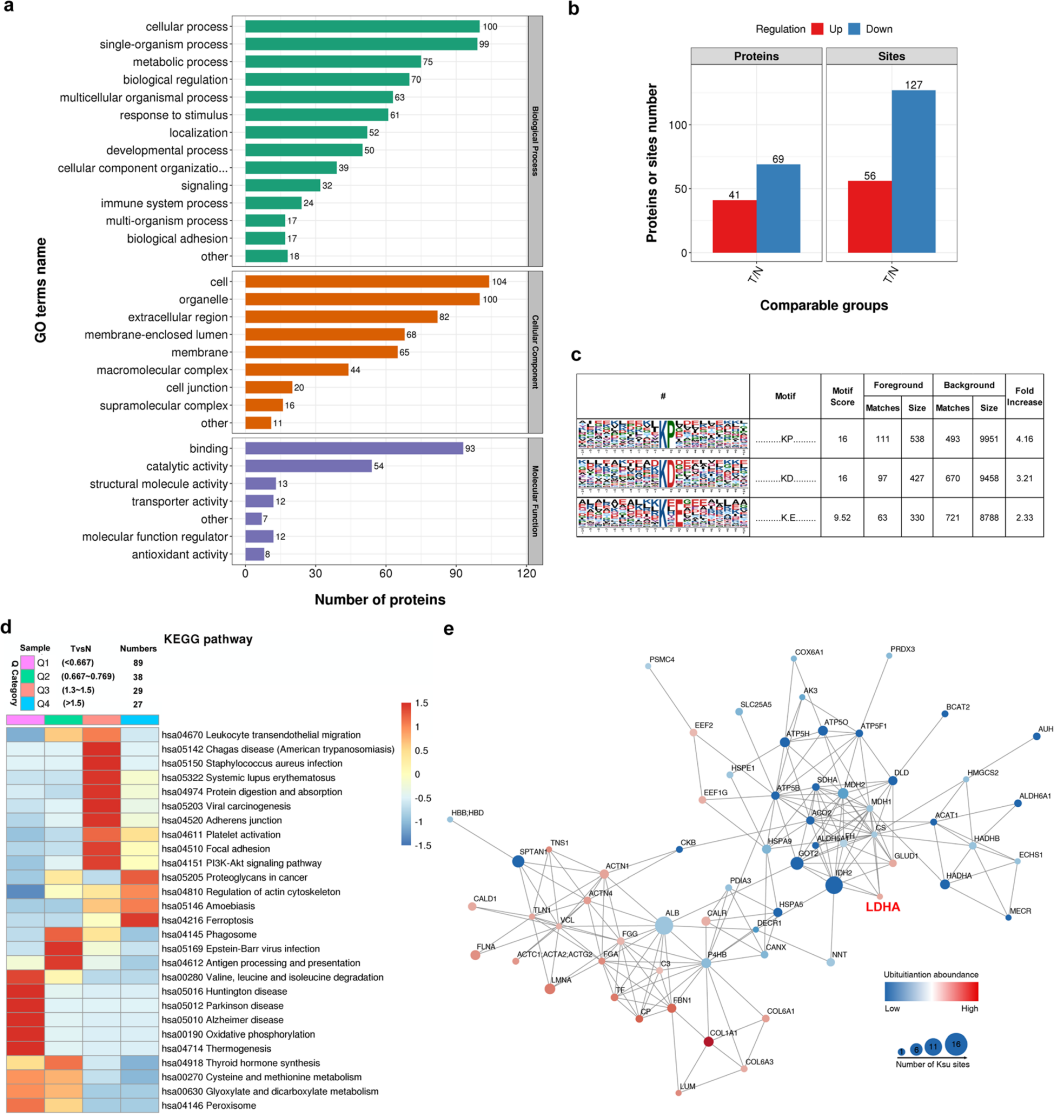
Proteomic quantification of lysine succinylation in human GC. **a** GO enrichment of quantified differentially expressed proteins. **b** Summary of differentially quantified sites and proteins (> 1.3 or < 0.77). **c** Motif analysis of all the identified succinylation sites. **d** KEGG pathway enrichment analysis of four groups of samples, Q1 (< 0.667), Q2 (0.667 ~ 0.769), Q3 (1.3 ~ 1.5), and Q5 (> 1.5) (Tumor-vs-Normal). **e** The protein-protein interaction (PPI) network analysis of differentially quantified sites and proteins (> 1.3 or < 0.77)

In order to study the effect of protein PTM in GC, we selected seven patients with primary GC who had not received any treatment before surgery, and surgically extracted their tumor tissues and adjacent normal tissues for the study. Here, we sought to analyze protein succinylation in GC samples. The tumor tissues and adjacent normal tissues of seven patients were digested and dissociated by trypsin. Using TMT labeling and affinity enrichment followed by high-resolution LC-MS/MS analysis, quantitative lysine succinylome analysis was performed. The mass spectrometry proteomics data have been deposited to the ProteomeXchange Consortium (http://proteomecentral.proteomexchange.org) via the iProX partner repository [27] with the dataset identifier PXD018376. Altogether, 503 lysine succinylation (Ksuc) sites in 303 proteins were identified, among which 331 sites in 188 proteins were quantified. Based on the quantification of lysine succinylation in tumor versus normal tissue, we set a ratio > 1.3 as upregulation and < 0.77 as downregulation thresholds. The identified modified proteins are found in various cell organelles and are involved in several metabolic pathways (Fig. 1a). The GO analysis of upregulated and downregulated proteins, biological processes, and cellular components involved are shown in Additional file 1 (Fig. S1a-d). Numerous differentially expressed proteins and Ksuc sites were obtained in each group, including 56 upregulated and 127 downregulated sites (Fig. 1b). Further, analysis of the succinylation sites revealed that the Ksuc motif showed a strong preference for proline (P) or aspartic acid (D) in the + 1 positions, as well as for glutamic acid (E) in the + 2 position (Fig. 1c). The samples were divided into four groups according to the quantitative ratio of lysine succinylation sites between tumor and adjacent normal tissues: Q1 (< 0.667), Q2 (0.667 ~ 0.769), Q3 (1.3 ~ 1.5), and Q5 (> 1.5). The results of KEGG pathway enrichment cluster analysis on the four groups are shown in Fig. 1d. Functions involved in biological processes and cellular components were also analyzed and are shown in Additional file 1 (Fig. S1c and d), respectively. Protein-protein interaction network analysis performed on all up- and downregulated proteins is shown in Fig. 1e.

### LDHA is up-regulated in GC and highly succinylated on K222

**Fig. 2:**
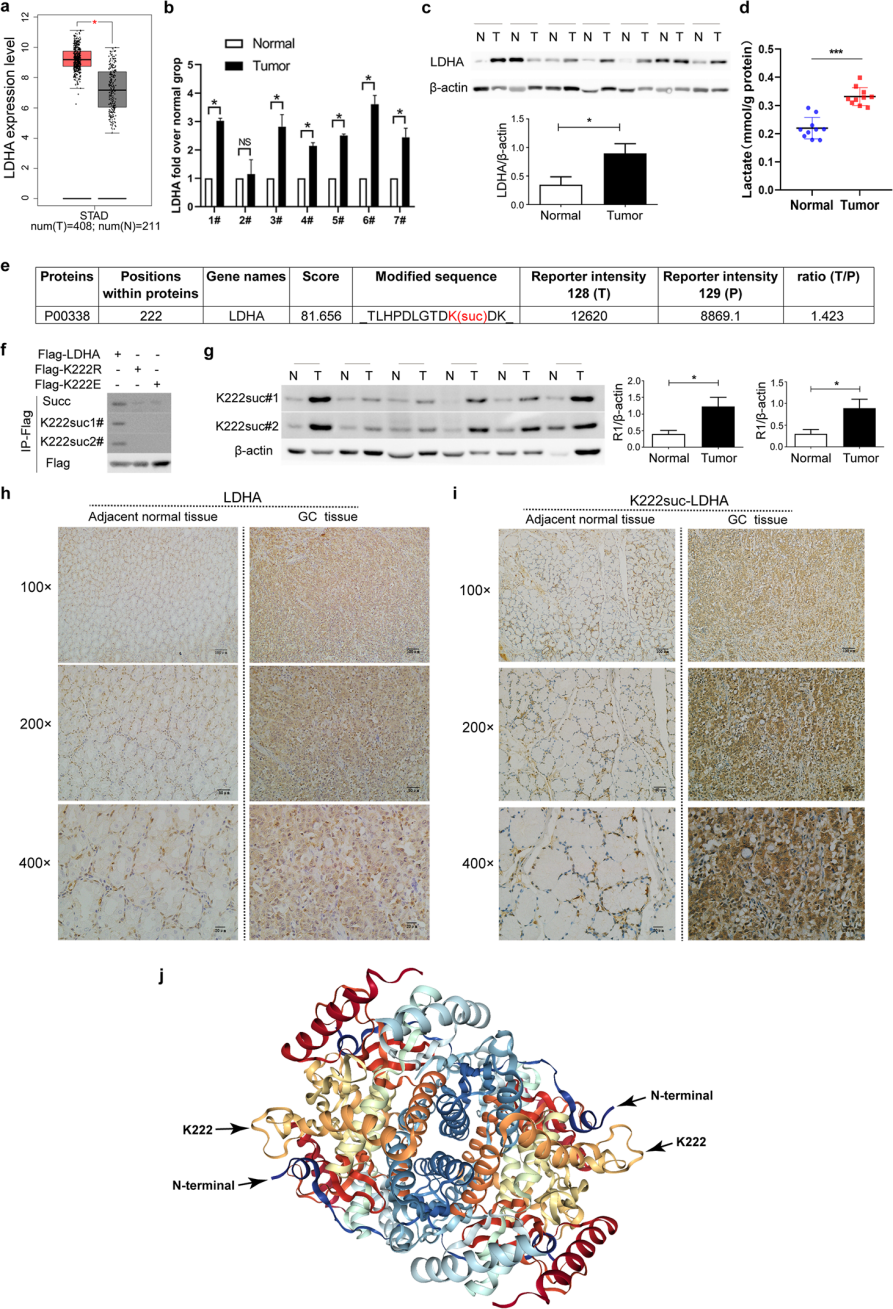
LDHA is highly expressed and succinylated at K222 in GC. **a** The gene expression of LDHA in GC samples (red) and paired normal tissues (green). Data from the GEPIA website. **b** Analysis of mRNA levels of LDHA in GC and adjacent normal tissues by qRT-PCR. **c** Analysis of the protein levels of LDHA in GC and adjacent normal tissues by western blot. **d** The lactate levels in GC and adjacent normal tissues. **e** Identification of succinylated LDHA peptides by mass spectrometry analysis of GC tissues. **f** K222 is the main succinylation site of LDHA. Cells (293 T) were transfected with the indicated plasmids. Then, immunoprecipitation (IP) was performed, followed by western blot analysis of succinylation. **g** Analysis of K222suc-LDHA levels in GC and adjacent normal tissues using two K222 succinylation-specific antibodies. **h** and **i** Immunohistochemical analysis of LDHA (**h**) and K222suc-LDHA (**i**) in GC and adjacent normal tissues. **j** The K222 site in the three-dimensional structure of the LDHA homotetramer. Data from the RCSB PBD, http://www.rcsb.org/3d-view/6MV8). Data are presented as mean ± SEM, *P < 0.05, ***P < 0.001

LDHA is highly expressed in several tumors, including GC (Fig. 2a), as showed on the GEPIA website (http://gepia.cancer-pku.cn/index.html) (Additional file 2: Fig. S2a, b), and is closely related to the prognosis and staging of tumors. Therefore, we compared the expression of LDHA in cancer and adjacent normal tissues of the seven patients. We found that the mRNA level of LDHA was significantly higher in cancer tissues than in adjacent normal tissues (Fig. 2b). Correspondingly, we found a significant increase in LDHA in tumor tissues at the protein level (Fig. 2c). Accordingly, the lactate level was significantly increased in tumor tissues than that in adjacent normal tissues (Fig. 2d). Mass spectrometry analysis showed that LDHA was succinylated on K222 in GC tissues, where the level of LDHA succinylation was 1.42 times higher than in adjacent normal tissues (Fig. 2e).

Next, we generated K222 succinylation-specific antibodies using a human K222-succinylated LDHA peptide (PDLGTDKsucDKEQWK) as the antigen. In addition, we synthesized and constructed the LDHA full-length plasmid (Flag-LDHA) and the mutant plasmids Flag-K222R (mimicking deletion) and Flag-K222E (mimicking the negatively charged succinyl-lysine modification). Both the pan anti-succinyl-lysine antibody and K222 succinylation-specific antibodies (#1 and #2) recognized the Flag-LDHA protein but not the K222 mutants (Fig. 2f). The level of K222suc-LDHA was significantly higher in GC tissues than in adjacent normal tissues, as indicated by western blot (Fig. 2g). Similarly, as shown by IHC, the levels of LDHA and K222suc-LDHA were higher in GC tissues than in adjacent normal tissues (Fig. 2h, i, respectively). However, the staining of K222suc-LDHA seems to be much stronger than that of LDHA (Fig. 2h, i).

Intriguingly, in the three-dimensional structure diagram of LDHA homotetramer (PDB: 6MV8, http://www.rcsb.org/3d-view/6MV8), K222 is highly exposed and extends outside (Fig. 2j). Succinylation at this site could therefore affect the binding capacity and protein-protein interactions, thereby influencing the structure and function of the protein.

### K222 succinylation does not affect the ubiquitination of LDHA

**Fig. 3:**
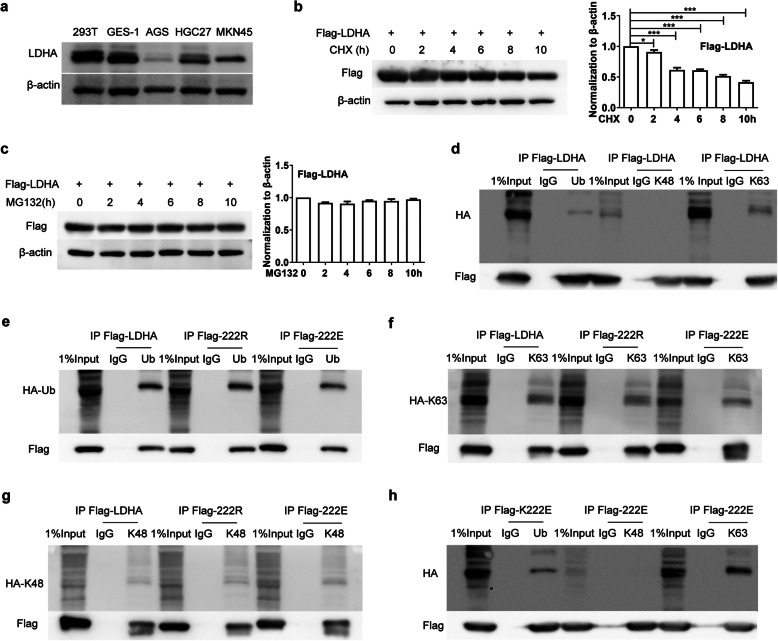
K222 succinylation does not affect the ubiquitination of LDHA. **a** The expression of LDHA in different cell lines. **b** and **c** LDHA is not degraded via the ubiquitin-proteasome pathway. Flag-LDHA was transfected into AGS cells for 24 h, then the cells were treated with CHX (10 μg/ml) (**b**) or MG132 (10 mM) (**c**) for the indicated time points. The cell lysates were collected and analyzed by western blot. **d** LDHA is mainly ubiquitinated on K63. AGS cells were transfected with the indicated plasmids and the IP was performed to pull down Flag-LDHA. The ubiquitination was detected by testing HA expression. **e**-**g** K222 mutation does not affect the ubiquitination of LDHA. AGS cells were transfected with indicated plasmids and the IP was performed to pull down Flag fusion proteins. The modification by ubiquitin (**e**), K63- (**f**) or K48- (**g**) ubiquitin was determined by HA antibody staining. **h** K222suc-LDHA is ubiquitinated and mainly modified by K63-ubiquitin. AGS cells were transfected with indicated plasmids and the co-IP was performed to pull down Flag-K222E protein. The ubiquitination was detected by measuring HA expression. Data are presented as mean ± SEM, *P < 0.05, ***P < 0.001

Next, the expression of LDHA was examined in 293 T cells and four GC cell lines. LDHA level was the lowest in AGS cells (Fig. 3a), indicating that these cells could be useful for exogenous LDHA expression. The main protein degradation pathways in the cell include the ubiquitin-proteasome system (UPS) and the autophagy-lysosome system. To investigate whether LDHA can be degraded upon ubiquitination, Flag-LDHA was overexpressed in AGS cells, which were then treated with protein synthesis inhibitor cycloheximide (CHX) or proteasome inhibitor MG132. After CHX treatment, Flag-LDHA expression decreased significantly over time (Fig. 3b). However, LDHA protein levels showed no obvious changes after treatment with MG132 (Fig. 3c). There are numerous ubiquitination sites in human LDHA protein, as shown on the Phosphosite website (https://www.phosphosite.org), which also include K222 site [28, 29]. To determine whether K222 succinylation affects the ubiquitination of LDHA, Flag-LDHA, Flag-K222R, or Flag-K222E in combination with HA-Ub (Ub), HA-Ub (K48), or HA-Ub (K63) plasmids were co-transfected into AGS cells. Flag-LDHA protein was pulled down by Flag antibody and ubiquitination was measured using HA antibody. The results showed that LDHA was ubiquitinated, mainly by K63-linked ubiquitin (Fig. 3d). K222 mutations, including the succinylation mimic K222E, did not affect the ubiquitination of LDHA (Fig. 3e-h).

### LDHA is degraded in lysosomes by binding to SQSTM1

**Fig. 4:**
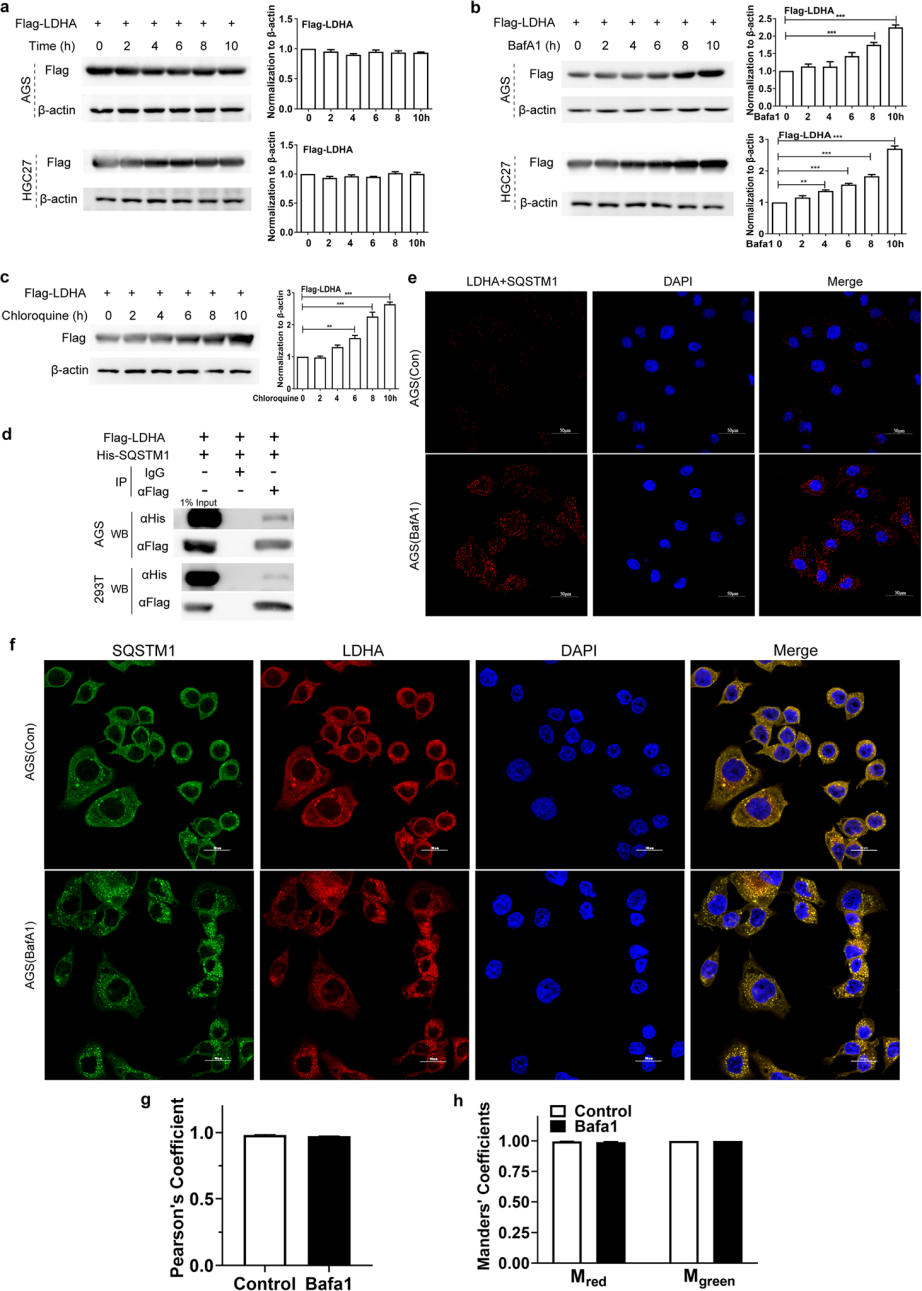
LDHA is targeted for lysosomal degradation by binding to SQSTM1. **a** Flag-LDHA is stably expressed in AGS or HGC27 cells after transfection. **b** and **c** LDHA is degraded by the lysosomal pathway. Flag-LDHA was transfected into AGS or HGC27 cells for 24 h, then the cells were treated with BafA1 (20 nM) (**b**) or chloroquine (50 μM) (**c**) for the indicated time points. The cell lysates were collected and analyzed by western blot. LDHA binds to SQSTM1. **d** Flag-LDHA interacts with His-SQSTM1 in AGS and 293 T cells. The cells were co-transfected with Flag-LDHA and His-SQSTM1. For the co-IP experiments, anti-FLAG antibody was used for pulldown and anti-His antibody for western blot. **e** PLA assay for detection of LDHA and SQSTM1 was performed in AGS cells treated with or without BafA1 (20 nM). **f** Colocalization of LDHA and SQSTM1 in AGS cells treated with or without BafA1 (20 nM) was observed by confocal microscopy. Magnification: 800x. The degree of colocalization was assessed using the Pearson’s coefficient (**g**) and the Mander’s coefficients (**h**). Data are presented as mean ± SEM, *P < 0.05, **P < 0.01, ***P < 0.001

Two inhibitors of the autophagy lysosomal pathway, chloroquine and Bafilomycin A1 (BafA1), were used to treat Flag-LDHA-overexpressing AGS or HGC27 cells. Flag-LDHA was expressed stably in AGS and HGC27 cells after transfection (Fig. 4a). Importantly, Flag-LDHA expression significantly increased after treatment with either BafA1 (Fig. 4b) or chloroquine (Fig. 4c). These results indicate that LDHA is indeed degraded by the autophagy lysosomal pathway. The ubiquitin proteasome pathway and autophagy lysosomal pathway are not completely independent from each other. There are a lot of cross-links between them such as sequestosome 1 (SQSTM1). Therefore, to investigate whether LDHA could interact with SQSTM1, AGS or 293 T cells were co-transfected with Flag-LDHA and His-SQSTM1 for 24 h prior to co-IP with either anti-Flag antibody or control IgG. Western blot analysis revealed that LDHA can interact with SQSTM1 (Fig. 4d). The interaction was further confirmed by in situ proximity ligation assay (PLA) (Fig. 3e). As shown in the figure, red PLA signals were visible in control-treated AGS cells (upper), but were much stronger in BafA1-treated AGS cells (lower). The co-localization of LDHA and SQSTM1 was also observed by immunofluorescence (magnification 800x in Fig. 4f) and indicated by the Pearson’s coefficient (Fig. 4g) and the Mander’s coefficients (Fig. 4h). In BafA1-treated AGS cells, Flag-LDHA expression in autophagy lysosomes significantly increased, and its localization considerably overlapped with that of SQSTM1, further demonstrating that LDHA is degraded by the lysosomal pathway.

### K222suc increases the levels of LDHA by reducing the binding of K63-ubiquitinated LDHA to SQSTM1

**Fig. 5:**
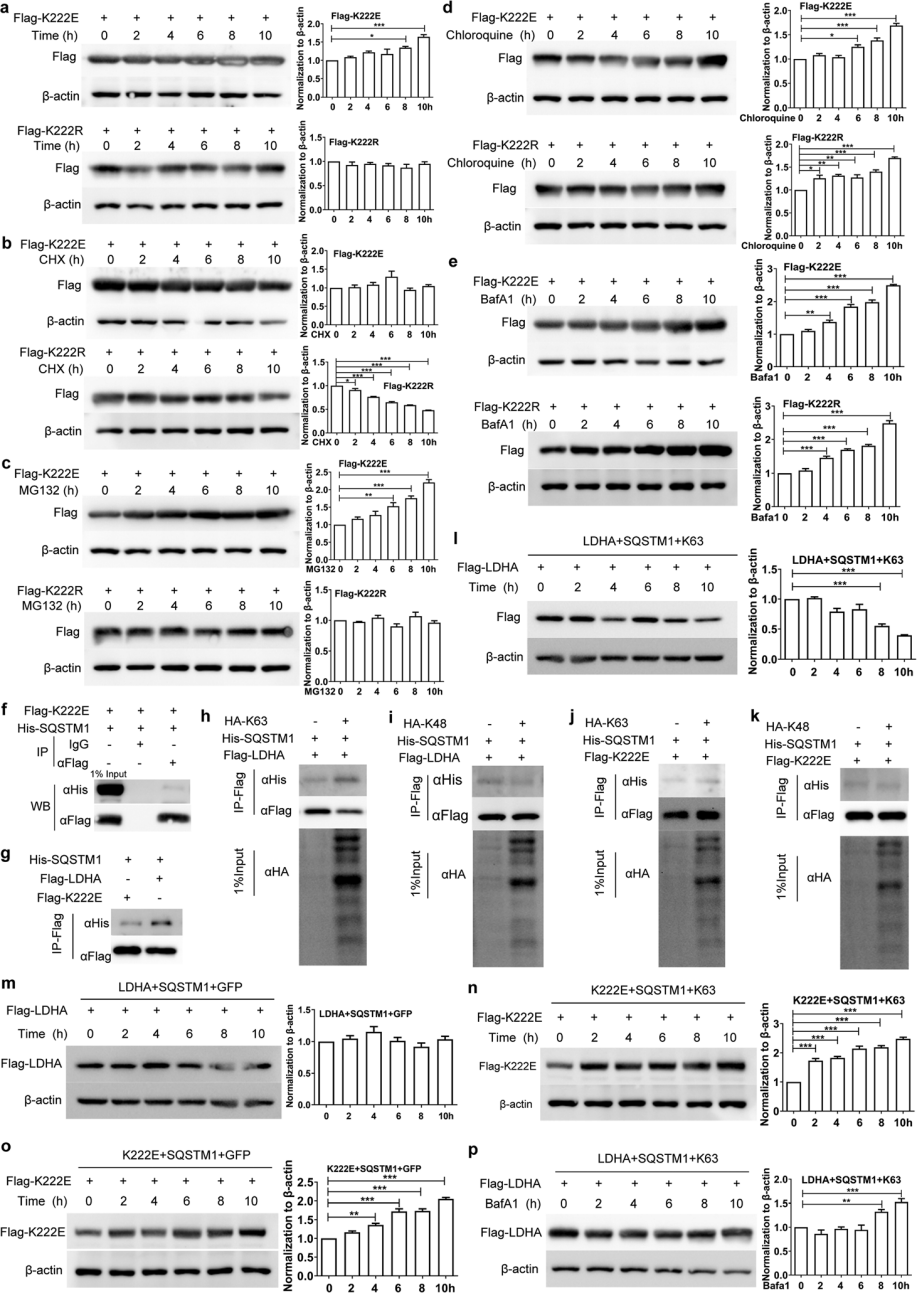
K222suc can decrease the binding of K63-ubiquitinated LDHA to SQSTM1, thereby reducing its degradation. **a** Flag-K222E (upper) or Flag-K222R (lower) fusion protein is stably expressed in AGS cells after transfection. **b**-**e** Flag-K222E protein was accumulated in cells and K222suc could inhibit the lysosomal degradation of LDHA. After transfection with Flag-K222E or Flag-K222R plasmid for 24 h, AGS cells were treated with CHX (10 μg/ml) (**b**), MG132 (10 mM) (**c**), chloroquine (50 μM) (**d**), or BafA1 (20 nM) (**e**). The cell lysates were collected at the indicated time points, and the K222E (upper) or Flag-K222R (lower) fusion protein was detected using anti-Flag antibody. **f** Flag-K222E binds to SQSTM1. **g** Succinylation of K222 decreases the binding of LDHA to SQSTM1. AGS cells were transfected with indicated plasmids, LDHA fusion proteins were immunoprecipitated by Flag antibody, and SQSTM1 protein was detected using anti-His antibody. **h** and **i** K63, rather than K48, ubiquitination promotes the interaction between LDHA and SQSTM1. AGS cells were transfected with Flag-LDHA and His-SQSTM1 with or without HA-K63 (H) or HA-K48 (I). Flag-LDHA was pulled down, and His-SQSTM1 was detected by western blot. **j** and **k** Neither K63 (**j**) nor K48 (**k**) ubiquitination can promote the interaction between Flag-K222E and SQSTM1. AGS cells were transfected with the indicated plasmids. Subsequently, Flag-K222E was immunoprecipitated, and His-SQSTM1 was detected by western blot. **l** and **m** K63 ubiquitination promotes the binding of LDHA to SQSTM1 leading to LDHA degradation. AGS cells were transfected with indicated plasmids for 24 h. Then, the cell lysates were collected at various time points, and Flag-LDHA was detected by western blot in the presence of K63-ubiquitin (**l**) or control GFP (**m**). **n** and **o** K63-linked ubiquitination did not promote the degradation of Flag-K222E. The experiments were performed as described in (**l**, **m**) but the Flag-K222E plasmid was used instead of Flag-LDHA. **p** BafA1 inhibited LDHA degradation promoted by K63 ubiquitination. Data are presented as mean ± SEM, *P < 0.05, **P < 0.01, ***P < 0.001

Exogenous Flag-K222E or Flag-K222R LDHA proteins were relatively stable in AGS or HGC27 cells after gene transfection (Fig. 5a, Fig. S3a). To investigate the effect of K222suc on the stability of LDHA, Flag-K222E or Flag-K222R plasmid-transfected cells were treated with CHX, MG132, chloroquine, or BafA1. Flag-K222E protein expression was relatively stable even after CHX treatment (Fig. 5b, upper; Fig. S3b), while that of Flag-LDHA (Fig. 3b) or Flag-K222R (Fig. 5b, lower) was significantly reduced. Interestingly, the addition of MG132 resulted in an increase in Flag-K222E protein levels (Fig. 5c, upper; Fig. S3c), but it did not affect the levels of either Flag-LDHA (Fig. 3c) or Flag-K222R (Fig. 5c, lower). However, treatment with chloroquine or BafA1 increased the protein levels of Flag-K222E (Fig. 5d, e, upper; Fig. S3d), Flag-LDHA (Fig. 4b, c), and Flag-K222R significantly (Fig. 5d, e, lower). These results suggest that Flag-K222E accumulated in cells and that K222suc could inhibit the lysosomal degradation of LDHA.

Consistently, Flag-K222E could still bind to SQSTM1 (Fig. 5f). However, the interaction of Flag-LDHA with SQSTM1 was much stronger than that of Flag-K222E (Fig. 5g). Furthermore, K63-linked ubiquitination promoted the binding of LDHA to SQSTM1 (Fig. 5h), while K48-linked ubiquitination had no such effect (Fig. 5i). Neither K63 nor K48 promoted the interaction between Flag-K222E and SQSTM1 (Fig. 5j, k, respectively). To further verify this, Flag-LDHA, His-SQSTM1, and either GFP or HA-UbK63 were co-transfected into AGS cells. Upon co-transfection of Flag-LDHA and SQSTM1 with K63, the protein level of Flag-LDHA decreased (Fig. 5l), while upon co-transfection with GFP, the protein level remained stable (Fig. 5m). Instead, the protein levels of Flag-K222E dramatically increased upon co-transfection of Flag-K222E and SQSTM1 with either K63 or GFP (Fig. 5n, o). In AGS cells co-transfected with Flag-LDHA, SQSTM1, and K63, Flag-LDHA accumulated upon treatment with BafA1 (Fig. 5p). These results show that K63-linked ubiquitination of LDHA leads to its degradation by promoting its binding to SQSTM1, whereas succinylation on K222 inhibits LDHA degradation by reducing its interaction with SQSTM1.

### CPT1A binds to and succinylates LDHA on K222

**Fig. 6:**
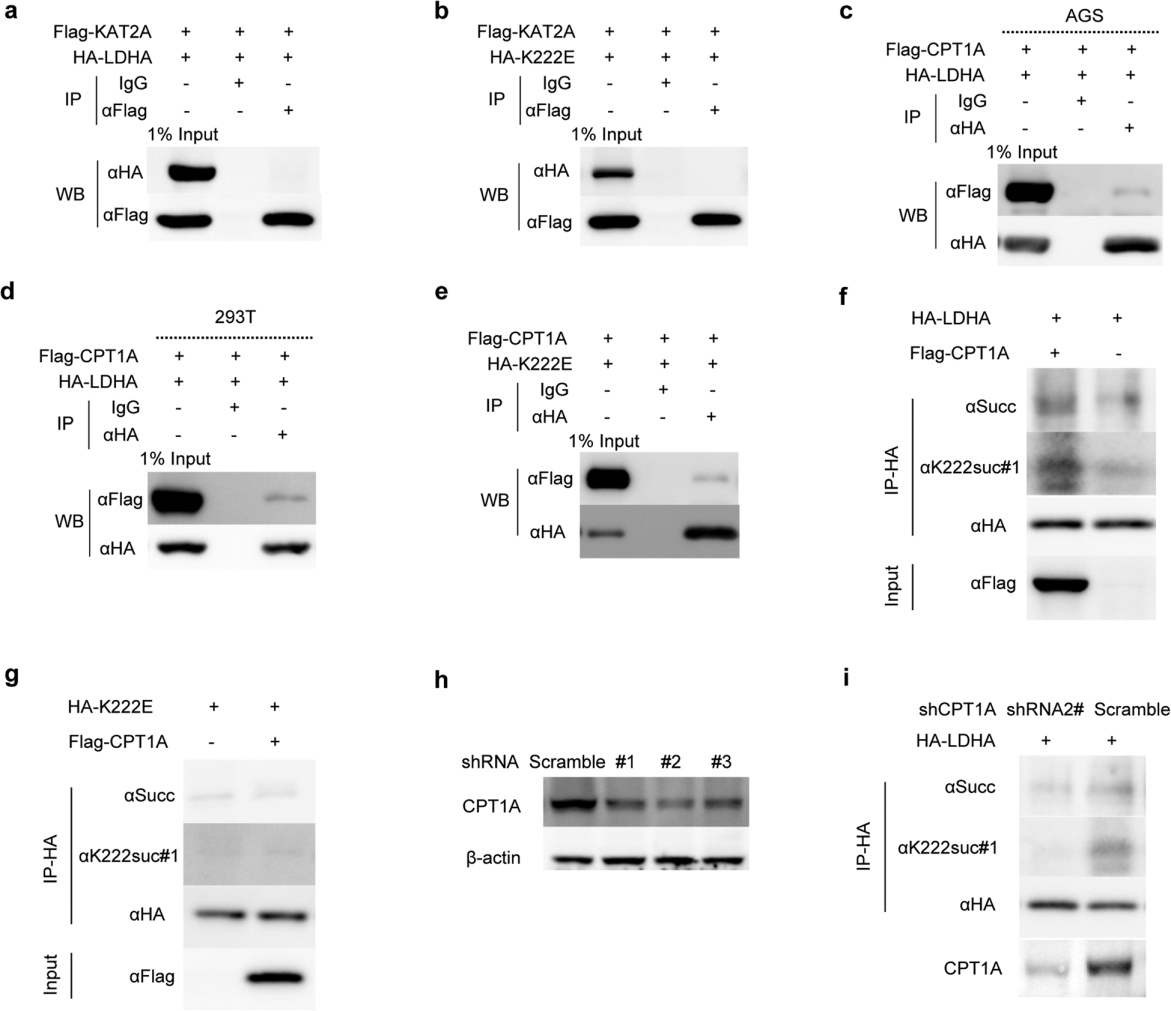
CPT1A binds to and succinylates LDHA at K222. **a** and **b** KAT2A does not bind to LDHA or its mutant K222E. AGS cells were transfected with Flag-KAT2A in combination with HA-LDHA or HA-K222E plasmid. KAT2A fusion protein was pulled down by Flag antibody, LDHA or K222E was detected using HA antibody. **c**-**e** CPT1A interacts with LDHA and its mutant K222E in AGS and 293 T cells. Cells were transfected with Flag-CPT1A in combination with HA-LDHA (**c**, **d**) or HA-K222E (**e**). The co-IP assay was performed similarly as above (**a**, **b**). **f** and **g** CPT1A promotes the succinylation of LDHA (**f**), but not of its mutant K222E (**g**). AGS cells were transfected with the indicated plasmids. Then, HA-LDHA was immunoprecipitated and its succinylation analyzed using either pan-succinylation or K222suc-specific antibody. **h** and **i** Knockdown efficiency of three CPT1A shRNAs in AGS cells was determined by western blot (**h**). CPT1A deficiency decreased the succinylation of LDHA (**i**)

Next, we sought to determine whether KAT2A and CPT1A could promote K222 succinylation of LDHA. KAT2A did not interact with either LDHA or K222E mutant (Fig. 6a, b). Remarkably, co-IP results indicated that CPT1A could bind to both LDHA (Fig. 6c, d) and K222E mutant (Fig. 6e). More importantly, overexpression of CPT1A increased the K222 succinylation of LDHA (Fig. 6f), but not of K222E mutant (Fig. 6g). To verify this effect, we knocked down CPT1A with three shRNAs, and chose the second shRNA based on its efficiency (Fig. 6h). Consequently, the knockdown of CPT1A resulted in the reduction of LDHA succinylation (Fig. 6i).

### K222-succinylated LDHA is associated with poor prognosis in patients with GC

**Fig. 7:**
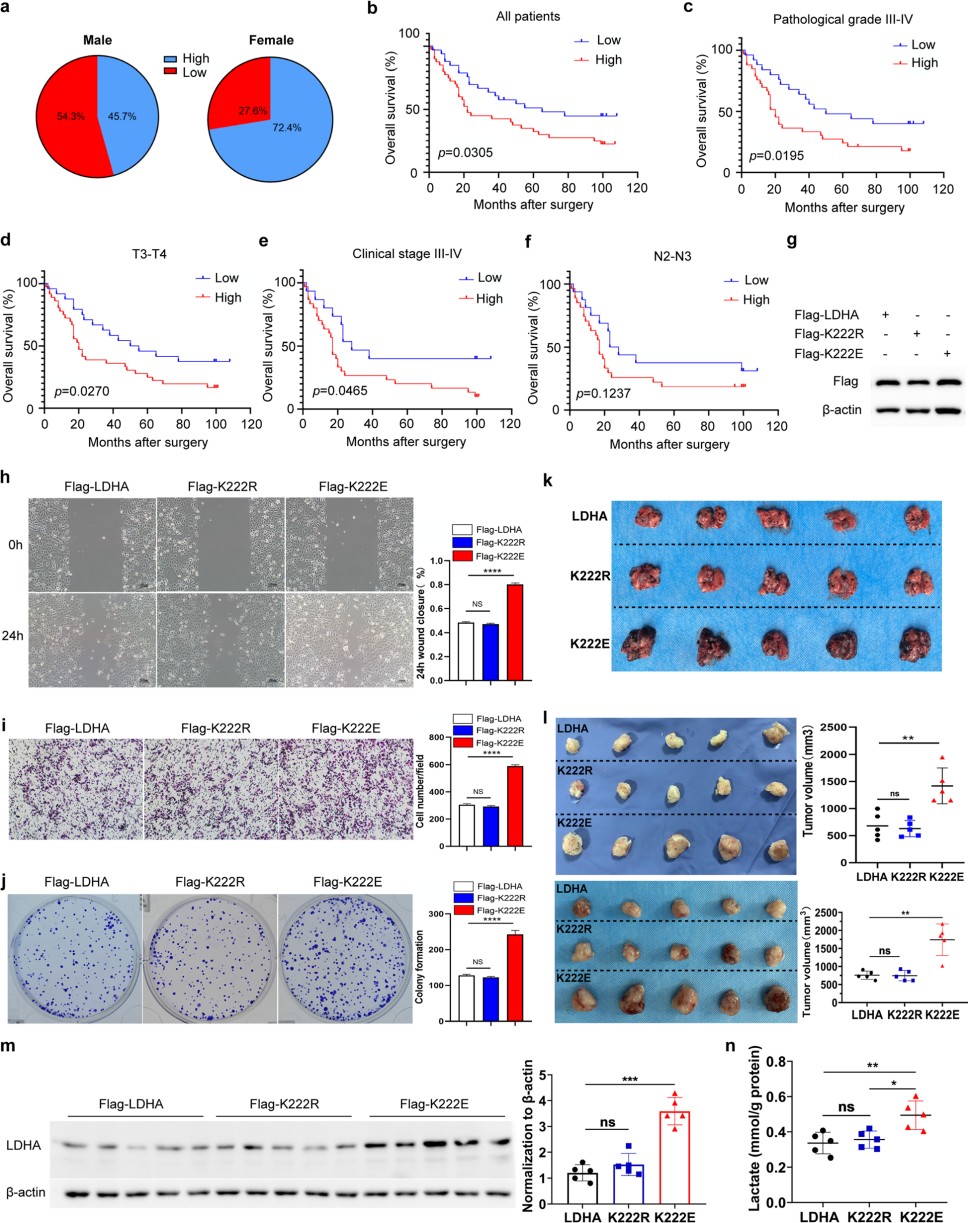
K222-succinylated LDHA is associated with poor prognosis in patients with GC, and succinylation-mimetic LDHA K222E mutant enhances cancer cell invasion and migration. **a** The proportion of LDHA with high K222 succinylation is higher in female than in male patients with GC. **b**-**f** Kaplan–Meier analysis of OS of patients with GC according to LDHA K222suc expression in all patients (**b**), patients with pathological grade III-IV tumors (**c**), patients with T3-T4 stage tumors (**d**), patients with clinical stage III-IV tumors (**e**), and patients with N2-N3 stage tumors (**f**). **g** Stable overexpression of Flag-LDHA, Flag-K222R, or Flag-K222E mutant was determined by western blot in AGS cells. **h** Wound-healing assay showed increased ability to migrate in AGS cells expressing Flag-K222E. **i** and **j** The transwell (**i**) and Plate colony formation assays (**j**) were performed to assess the invasion and proliferation of AGS cells. **k** Mouse model of B16-F10 lung metastasis, visible as black dots on the surface of lungs from three groups. **l** Succinylation mimetic LDHA K222E mutant facilitates tumor growth. AGS cells stably overexpressing Flag-LDHA, Flag-K222R, or Flag-K222E were injected subcutaneously in nude mice. Final tumors from two independent experiments were photographed, and tumor volumes were measured. **m** Analysis of the protein levels of LDHA in the subcutaneous xenografts from AGS cells in nude mice by western blot. **n** The lactate levels in the subcutaneous xenografts in nude mice from three groups. Date are presented as mean ± SEM; *P < 0.05, **P < 0.01, ***P < 0.001

To analyze the association of LDHA K222suc and prognosis of patients with GC, the specific K222suc antibody was employed to conduct tissue microarray-based IHC. Tumor tissues and adjacent normal tissues were scored according to the IHC results. K222suc was considered upregulated in samples with a ratio > 1.3 in tumor/normal tissue (Table 1). Interestingly, we found that the proportion of LDHA with high K222 succinylation was much higher in female patients than in male patients (Fig. 7a). Moreover, among all patients, those with high succinylation of LDHA K222 had lower overall survival (OS) (Fig. 7b). We also analyzed the OS based on other key clinicopathological factors and according to LDHA K222suc levels. Significant differences were found in pathological grade III-IV, T3-T4, and clinical stage III-IV tumors (Fig. 7c-e). Interestingly, although the tumors with higher LDHA K222 succinylation were more likely to metastasize to lymph nodes (Table 1), the OS did not significantly differ in patients with a higher degree of lymphatic metastasis (N2-N3) (Fig. 7f).

**Table 1:** Associations between K222suc-LDHA expression and clinical pathological characteristics in patients with GC

Variable	n	Low (K222suc)	High (K222suc)	***P*** value
Age				0.8090
≤ 60	26	12	14	
> 60	47	20	27	
Gender				0.0318
Male	46	25	21	
Female	29	8	21	
Pathological grade				0.0395
I-II	21	13	8	
III-IV	55	19	36	
Depth of invasion (T)				0.0125
T1-T2	17	12	5	
T3-T4	60	21	39	
Lymph node metastasis				0.0358
N0-N1	32	18	14	
N2-N3	45	14	31	
Distant metastasis (M)				
Negative (M0)	67	27	40	
Positive (M1)	9	5	4	
Clinical stage				0.0318
I-II	30	18	12	
III-IV	42	14	28

### LDHA K222suc promotes the migration and proliferation of GC cells

To explore the effect of LDHA K222suc on the function of GC cells, we established AGS cells stably expressing Flag-LDHA, Flag-K222R, and Flag-K222E using lentiviruses (Fig. 7g). As determined by wound-healing assays, AGS cells expressing Flag-K222E had increased ability to migrate (Fig. 7h). Similarly, transwell and plate colony formation experiments showed that invasion and proliferation also increased in AGS cells expressing Flag-K222E, but did not significantly change in cells expressing Flag-LDHA and Flag-K222R (Fig. 7i, j). To address the biological significance of K222 succinylation in vivo, we performed mouse melanoma lung metastasis experiments using B16-F10 cell lines overexpressing WT, K222R or K222E mutant LDHA. Our data show that K222E mutant LDHA-overexpressing B16-F10 cells caused more lung metastasis than WT or K222R LDHA-overexpressing cells (Fig. 7k).

Furthermore, a xenograft model was adopted to determine the effect of Flag-K222E on tumor growth. We inoculated AGS cells stably expressing Flag-LDHA, Flag-K222R, and Flag-K222E subcutaneously in nude mice to observe the formation and the size of tumors. AGS cells expressing Flag-K222E formed larger subcutaneous tumors than AGS cells expressing Flag-LDHA or Flag-K222R, and there was no significant difference between the tumors from AGS cells expressing Flag-LDHA and Flag-K222R (Fig. 7l). Moreover, the level of LDHA and lactate were significantly higher in the tumors from Flag-K222E group as shown in Fig. 7m and n, respectively. Together, these data indicated that K222E, which mimics K222suc, promoted tumor cell invasion, metastasis, and growth.

## Discussion

PTM is one of the two main mechanisms that expand the diversity of proteins, along with mRNA splicing, which occurs at the transcriptional level. Lysine is a typical target of modifications, and it has a positive charge under physiological conditions [6, 7]. Succinylation and acetylation of lysine have many similarities but also essential differences. Acetylation can change the group charge of lysine from + 1 to 0 [30, 31]. Instead, lysine succinylation can change the charge of two groups from + 1 to − 1, which is similar to the change of two groups from 0 to − 2 induced by phosphorylation [32]. Moreover, the succinyl groups are much larger than the acetyl groups [6]. Succinylation of lysine can therefore induce profound changes in the modified proteins, indicating that, like acetylation and phosphorylation, this modification could also play an important role in cell metabolism and function.

Lactate dehydrogenase mainly consists of two different subunits, LDHA (M subunit) and LDHB (H subunit), catalyzing the reversible conversion of pyruvate to lactic acid accompanied by conversion of NADH to NAD^+^ and ATP. The LDHA subtype is mainly expressed in skeletal muscle, where it gives priority to the conversion of pyruvate to lactic acid, while the LDHB subtype is mainly expressed in the heart and brain, where it prioritizes the conversion of lactic acid to pyruvate [16, 17]. LDHA is the key enzyme in the Warburg effect, which is a hallmark of cancer. The known PTMs of LDHA include acetylation, phosphorylation, mono-methylation, succinylation, sumoylation, and ubiquitylation, which are shown in Table S1 (from PhosphoSitePlus website, https://www.phosphosite.org/siteTableNewAction?id=4103&showAllSites=true). Acetylation of LDHA at K5 inhibits its activity and increases its interaction with HSP70, thereby promoting its degradation [33]. Additionally, phosphorylation of LDHA at Y10 mediated by human epidermal growth factor receptor 2 (HER2) and avian sarcoma viral oncogene v-src homolog (Src) enhances the formation of LDHA tetramers, increases its activity, and provides an anti-anoikis, pro-invasive, and pro-metastatic potential to cancer cells [34]. In this study, we found that LDHA is succinylated at K222, and that LDHA succinylation is significantly higher in GC tissues than in adjacent normal tissues, which is also closely related to the high expression of LDHA in GC.

The UPS and autophagy-lysosome system are involved in the degradation of most cellular proteins and maintain protein homeostasis [35, 36]. The two systems have long been considered as independent mechanisms of protein degradation. However, a growing number of studies have found several cross-links between them. When the UPS is inhibited, the autophagy-lysosome system can isolate the accumulated abnormal proteins in autophagosomes and deliver them to lysosomes for degradation. Conversely, the autophagy-lysosome system damage also leads to UPS up-regulation. Previously, autophagy was thought to be a non-selective degradation process. It is now known that autophagy can specifically degrade proteins through autophagy receptors. As a characteristic autophagy receptor, SQSTM1 (also known as p62) plays an important role in clearing aggregate proteins and pathogens, and it is a substrate for autophagy itself [37]. Moreover, SQSTM1 can also function as a ubiquitination receptor, bringing ubiquitinated proteins to the autophagy lysosome pathway for degradation [35, 38].

It has been reported that LDHA is mainly degraded through the lysosomal pathway, and is targeted for degradation by binding to HSPs [33]. Interestingly, LDHA can also be ubiquitinated, mainly with K63-linked ubiquitin. However, after treating tumor cells with MG132, chloroquine, or BafA1, we found that LDHA is not degraded by the UPS. In fact, LDHA K63 ubiquitination promotes the binding of LDHA to SQSTM1, therefore leading LDHA to the lysosomes for degradation.

Moreover, following LDHA K222 succinylation, the binding of LDHA with SQSTM1 is significantly reduced, although its K63-linked ubiquitination is not affected. Lysine has a positive charge at physiological pH (7.4). The URB box and ZZ-domain of SQSTM1 utilize their negatively charged pocket to recognize and bind with positively charged protein-type 1 N-end rule substrates, including lysine, to promote protein lysosomal degradation [39]. We observed that the K222 site is completely exposed and extremely close to the N-terminus of LDHA (Fig. 2i). It is predictable that the succinylation of K222 results in a change in the electric charge of K222 from + 1 to − 1, which could abrogate the interaction between LDHA and SQSTM1. Meanwhile, we demonstrated that CPT1A, not KAT2A, functions as the lysine succinyltransferase that binds to and regulates the succinylation of LDHA.

LDHA is becoming a promising new target in cancer therapy due to its high expression and correlation to poor prognosis in tumors. However, LDHA is ubiquitously expressed in cells, including non-cancerous cells. Moreover, LDHA gene knockout is fatal in mice. Therefore, reducing the level of LDHA selectively in tumor cells without affecting normal cell function remains a major challenge. Encouragingly, our data from tissue microarray showed that K222-succinylated LDHA was closely related to survival and prognosis of patients. Notably, the proportion of women with GC expressing succinylated LDHA is higher than that of men. Consequently, the succinylated LDHA is probably the better choice. Intriguingly, LDHA K222E missense mutant, a fast-type electrophoretic variant was discovered in a female patient with chest pain as well as her son, where LDHA activity in serum was within the normal reference interval [40]. Whether and how LDHA K222E missense mutation occurs in patients with GC warrant an in-depth investigation. In addition, transgenic mice with targeted LDHA K222E mutation need to be constructed and applied in murine models of cancer to further explore the role of K222E mutation.

## Conclusions

In summary, our data revealed a novel lysosomal pathway of LDHA degradation, which is mediated by the binding of K63-ubiquitinated LDHA to SQSTM1. Strikingly, CPT1A succinylates LDHA on K222, which thereby reduces the binding and inhibits the degradation of LDHA, as well as promotes GC invasion and proliferation. Notably, K222-succinylated LDHA is associated with poor prognosis in patients with GC. Therefore, targeting succinylated LDHA provides a novel strategy for GC therapy.

## Supplementary information

**Additional file 1: Fig. S1.** Proteomic quantification of lysine succinylation analysis in human GC. **a-d** GO-based enrichment analysis of up-regulated (**a**) and down-regulated (**b**) proteins (Tumour-vs-Normal). The biological process (**c**) and cellular component (**d**) analysis of the distribution of quantification results.

**Additional file 2: Fig. S2.** The expression of LDHA in various types of tumors. **a** and **b** The gene expression profile across all tumor samples (red) and paired normal tissues (green). Each dots represent expression of samples.

**Additional file 3: Fig. S3.** K222suc reduces the degradation of LDHA in HGC27 cells. **a** Flag-K222E (K222suc mimic) fusion protein is stably expressed in HGC27 cells after transfection. **b**-**d** Flag-K222E protein was accumulated in HGC27 cells and K222suc could inhibit the lysosomal degradation of LDHA. After transfection with Flag-K222E plasmid for 24 h, HGC27 cells were treated with CHX (10 μg/ml) (**b**), MG132 (10 mM) (**c**), or BafA1 (20 nM) (**d**). The cell lysates were collected at the indicated time points, and the K222E fusion protein was detected using anti-Flag antibody. Data are presented as mean ± SEM, *P < 0.05, **P < 0.01, ***P < 0.001.

**Additional file 4: Table S1.** PTM sites in human LDHA (PhosphoSitePlus).

## Data Availability

The data supporting the findings of this study are included in this paper and its additional files. The mass spectrometry proteomics data have been deposited to the ProteomeXchange Consortium (http://proteomecentral.proteomexchange.org) with the dataset identifier PXD018376.

## Abbreviations

GC: Gastric cancer
PTM: Protein posttranslational modification
LDHA: Lactate dehydrogenase A
SQSTM1: Sequestosome 1
BafA1: Bafilomycin A1
CHX: Cycloheximide
CPT1A: Carnitine Palmitoyltransferase 1A
KAT2A: Lysine acetyltransferase 2A
co-IP: Co-immunoprecipitation
PLA: Proximity ligation assay
LDHA K222suc: The succinylation of LDHA at lysine 222

## References

[1] Walsh CT, Garneau-Tsodikova S, Gatto GJ (2005). Protein posttranslational modifications: the chemistry of proteome diversifications. Angew Chem Int Ed Engl.

[2] Chen Y, Sprung R, Tang Y, Ball H, Sangras B, Kim SC (2007). Lysine propionylation and butyrylation are novel post-translational modifications in histones. Mol Cell Proteomics.

[3] Zhang K, Chen Y, Zhang Z, Zhao Y (2009). Identification and verification of lysine propionylation and butyrylation in yeast core histones using PTMap software. J Proteome Res.

[4] Zhang D, Tang Z, Huang H, Zhou G, Cui C, Weng Y (2019). Metabolic regulation of gene expression by histone lactylation. Nature.

[5] Hasan MM, Khatun MS, Kurata H: Large-Scale Assessment of Bioinformatics Tools for Lysine Succinylation Sites. Cells. 2019;8(2):95. 10.3390/cells8020095 PMC6406724 30696115

[6] Zhang Z, Tan M, Xie Z, Dai L, Chen Y, Zhao Y (2011). Identification of lysine succinylation as a new post-translational modification. Nat Chem Biol.

[7] Alleyn M, Breitzig M, Lockey R, Kolliputi N (2018). The dawn of succinylation: a posttranslational modification. Am J Physiol Cell Physiol.

[8] Wang G, Meyer JG, Cai W, Softic S, Li ME, Verdin E (2019). Regulation of UCP1 and Mitochondrial Metabolism in Brown Adipose Tissue by Reversible Succinylation. Mol Cell.

[9] Qi H, Ning X, Yu C, Ji X, Jin Y, McNutt MA (2019). Succinylation-dependent mitochondrial translocation of PKM2 promotes cell survival in response to nutritional stress. Cell Death Dis.

[10] Wang Y, Guo YR, Liu K, Yin Z, Liu R, Xia Y (2017). KAT2A coupled with the alpha-KGDH complex acts as a histone H3 succinyltransferase. Nature.

[11] Kurmi K, Hitosugi S, Wiese EK, Boakye-Agyeman F, Gonsalves WI, Lou Z (2018). Carnitine Palmitoyltransferase 1A has a lysine Succinyltransferase activity. Cell Rep.

[12] Wang Y, Guo YR, Xing D, Tao YJ, Lu Z (2018). Supramolecular assembly of KAT2A with succinyl-CoA for histone succinylation. Cell Discov.

[13] Wang C, Zhang C, Li X, Shen J, Xu Y, Shi H (2019). CPT1A-mediated succinylation of S100A10 increases human gastric cancer invasion. J Cell Mol Med.

[14] Mishra D, Banerjee D: Lactate Dehydrogenases as Metabolic Links between Tumor and Stroma in the Tumor Microenvironment. Cancers (Basel). 2019;11(6):750. 10.3390/cancers11060750 PMC6627402 31146503

[15] Warburg O (1956). On the origin of cancer cells. Science.

[16] Kolappan S, Shen DL, Mosi R, Sun J, McEachern EJ, Vocadlo DJ (2015). Structures of lactate dehydrogenase a (LDHA) in apo, ternary and inhibitor-bound forms. Acta Crystallogr D Biol Crystallogr.

[17] Zdralevic M, Brand A, Di Ianni L, Dettmer K, Reinders J, Singer K (2018). Double genetic disruption of lactate dehydrogenases a and B is required to ablate the “Warburg effect” restricting tumor growth to oxidative metabolism. J Biol Chem.

[18] Das CK, Parekh A, Parida PK, Bhutia SK, Mandal M (2019). Lactate dehydrogenase a regulates autophagy and tamoxifen resistance in breast cancer. Biochim Biophys Acta Mol Cell Res.

[19] Dorneburg C, Fischer M, Barth TFE, Mueller-Klieser W, Hero B, Gecht J (2018). LDHA in neuroblastoma is associated with poor outcome and its depletion decreases neuroblastoma growth independent of aerobic glycolysis. Clin Cancer Res.

[20] Zhao J, Huang X, Xu Z, Dai J, He H, Zhu Y (2017). LDHA promotes tumor metastasis by facilitating epithelialmesenchymal transition in renal cell carcinoma. Mol Med Rep.

[21] Ping W, Senyan H, Li G, Yan C, Long L (2018). Increased lactate in gastric Cancer tumor-infiltrating lymphocytes is related to impaired T cell function due to miR-34a deregulated lactate dehydrogenase a. Cell Physiol Biochem.

[22] Liu Y, Guo JZ, Liu Y, Wang K, Ding W, Wang H (2018). Nuclear lactate dehydrogenase a senses ROS to produce alpha-hydroxybutyrate for HPV-induced cervical tumor growth. Nat Commun.

[23] Le A, Cooper CR, Gouw AM, Dinavahi R, Maitra A, Deck LM (2010). Inhibition of lactate dehydrogenase a induces oxidative stress and inhibits tumor progression. Proc Natl Acad Sci U S A.

[24] Feng Y, Xiong Y, Qiao T, Li X, Jia L, Han Y (2018). Lactate dehydrogenase a: a key player in carcinogenesis and potential target in cancer therapy. Cancer Med.

[25] Hameed O, Chhieng DC, Adams AL (2007). Does using a higher cutoff for the percentage of positive cells improve the specificity of HER-2 immunohistochemical analysis in breast carcinoma?. Am J Clin Pathol.

[26] Geng B, Pan J, Zhao T, Ji J, Zhang C, Che Y (2018). Chitinase 3-like 1-CD44 interaction promotes metastasis and epithelial-to-mesenchymal transition through beta-catenin/Erk/Akt signaling in gastric cancer. J Exp Clin Cancer Res.

[27] Ma J, Chen T, Wu S, Yang C, Bai M, Shu K (2019). iProX: an integrated proteome resource. Nucleic Acids Res.

[28] Akimov V, Barrio-Hernandez I, Hansen SVF, Hallenborg P, Pedersen AK, Bekker-Jensen DB (2018). UbiSite approach for comprehensive mapping of lysine and N-terminal ubiquitination sites. Nat Struct Mol Biol.

[29] Mertins P, Qiao JW, Patel J, Udeshi ND, Clauser KR, Mani DR (2013). Integrated proteomic analysis of post-translational modifications by serial enrichment. Nat Methods.

[30] Yang XJ, Seto E (2008). Lysine acetylation: codified crosstalk with other posttranslational modifications. Mol Cell.

[31] Guan KL, Xiong Y (2011). Regulation of intermediary metabolism by protein acetylation. Trends Biochem Sci.

[32] Sadhukhan S, Liu X, Ryu D, Nelson OD, Stupinski JA, Li Z (2016). Metabolomics-assisted proteomics identifies succinylation and SIRT5 as important regulators of cardiac function. Proc Natl Acad Sci U S A.

[33] Zhao D, Zou SW, Liu Y, Zhou X, Mo Y, Wang P (2013). Lysine-5 acetylation negatively regulates lactate dehydrogenase a and is decreased in pancreatic cancer. Cancer Cell.

[34] Jin L, Chun J, Pan C, Alesi GN, Li D, Magliocca KR (2017). Phosphorylation-mediated activation of LDHA promotes cancer cell invasion and tumour metastasis. Oncogene.

[35] Meyer-Schwesinger C (2019). The ubiquitin-proteasome system in kidney physiology and disease. Nat Rev Nephrol.

[36] Milan E, Perini T, Resnati M, Orfanelli U, Oliva L, Raimondi A (2015). A plastic SQSTM1/p62-dependent autophagic reserve maintains proteostasis and determines proteasome inhibitor susceptibility in multiple myeloma cells. Autophagy.

[37] Deng Z, Purtell K, Lachance V, Wold MS, Chen S, Yue Z (2017). Autophagy receptors and neurodegenerative diseases. Trends Cell Biol.

[38] Wang Z, Zhang H (2019). Phase separation, transition, and Autophagic degradation of proteins in development and pathogenesis. Trends Cell Biol.

[39] Kwon DH, Park OH, Kim L, Jung YO, Park Y, Jeong H (2018). Insights into degradation mechanism of N-end rule substrates by p62/SQSTM1 autophagy adapter. Nat Commun.

[40] Maekawa M, Sudo K, Kobayashi A, Sugiyama E, Li SS, Kanno T (1994). Fast-type electrophoretic variant of lactate dehydrogenase M(a) and comparison with other missense mutations in lactate dehydrogenase M(a) and H(B) genes. Clin Chem.

