# Supplementary figures and images for: Lysine-222 succinylation reduces lysosomal degradation of lactate dehydrogenase a and is increased in gastric cancer
Source: J Exp Clin Cancer Res. 2020 Aug 28;39:172. doi: 10.1186/s13046-020-01681-0 (PMC7455916; doi:10.1186/s13046-020-01681-0)

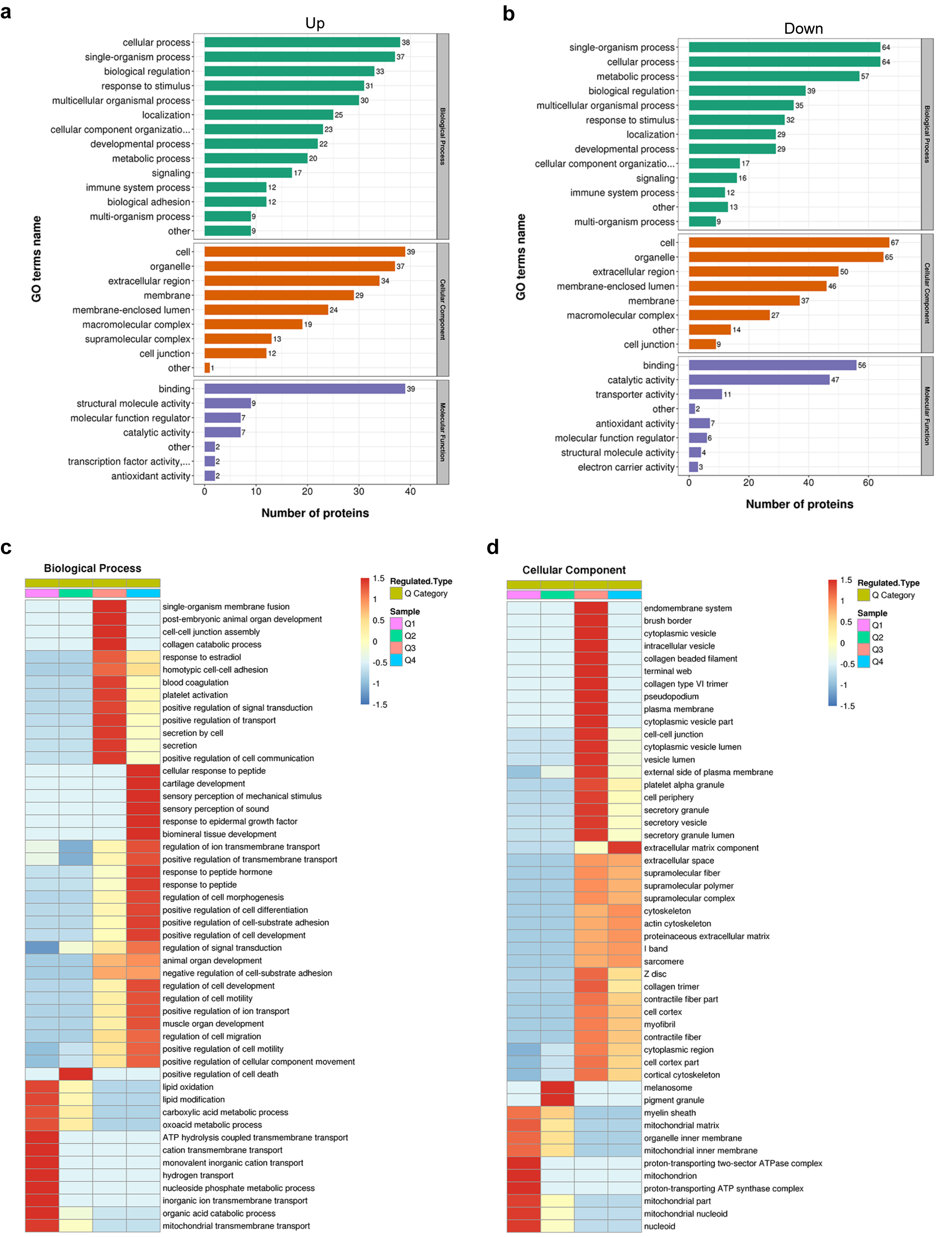

Supplement: Supplementary file 1 — Additional file 1: Fig. S1. Proteomic quantification of lysine succinylation analysis in human GC. a-d GO-based enrichment analysis of up-regulated (a) and down-regulated (b) proteins (Tumour-vs-Normal). The biological process (c) and cellular component (d) analysis of the distribution of quantification results. [file 13046_2020_1681_MOESM1_ESM.tif]

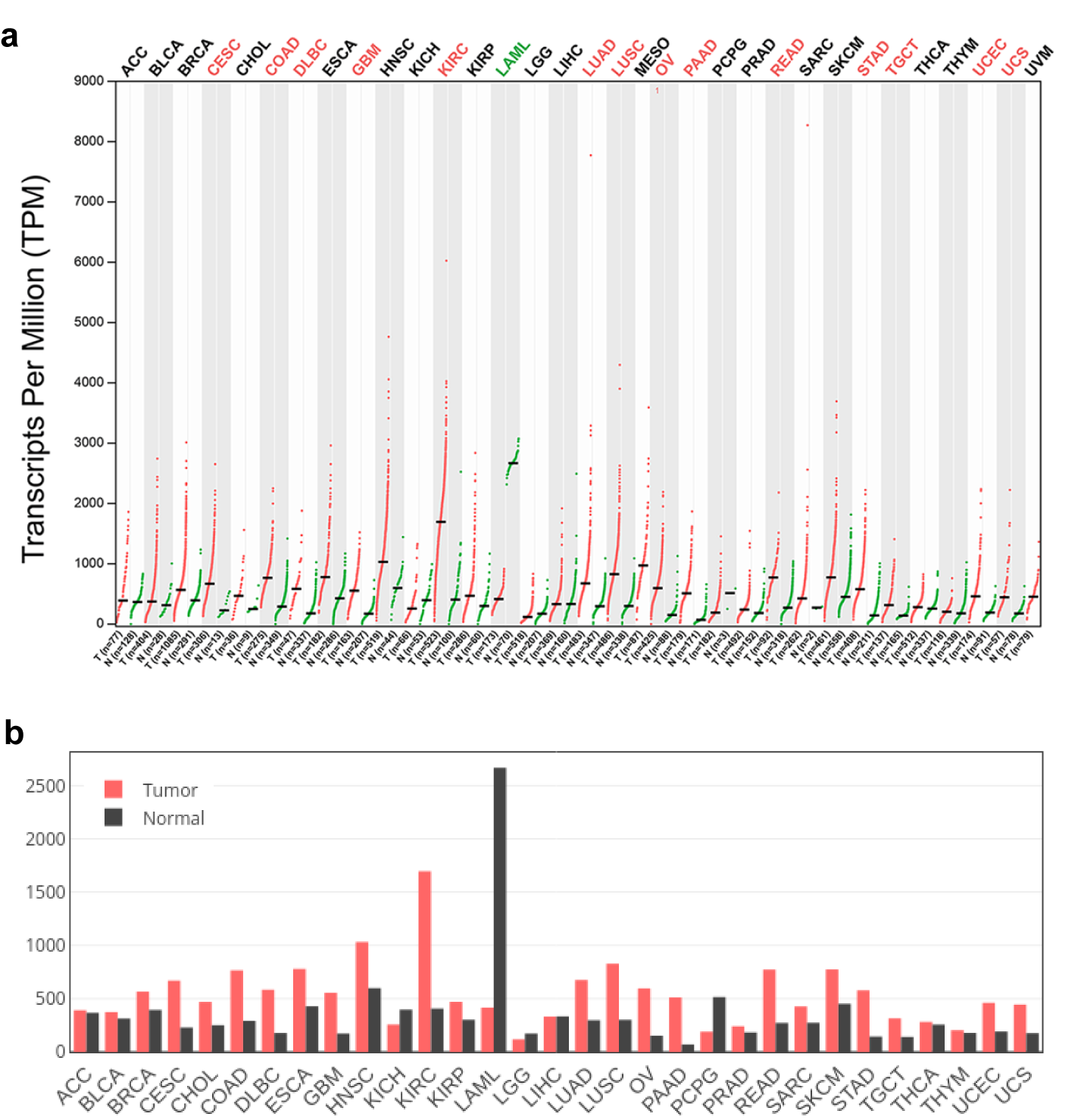

Supplement: Supplementary file 2 — Additional file 2: Fig. S2. The expression of LDHA in various types of tumors. a and b The gene expression profile across all tumor samples (red) and paired normal tissues (green). Each dots represent expression of samples. [file 13046_2020_1681_MOESM2_ESM.tif]

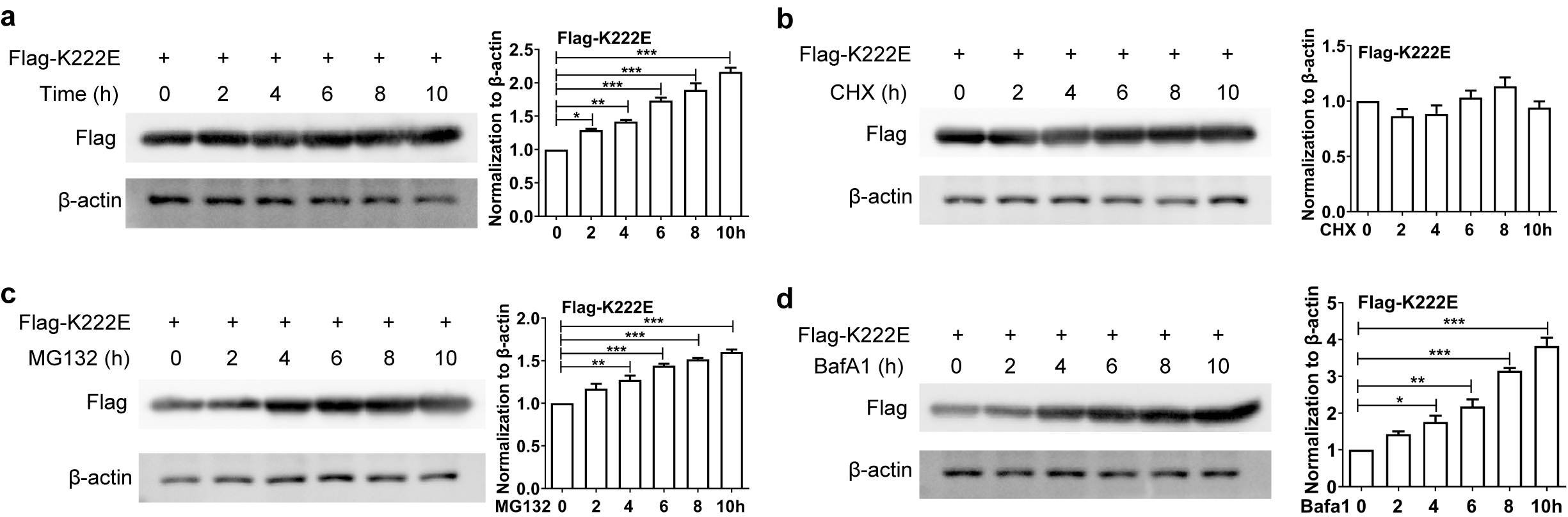

Supplement: Supplementary file 3 — Additional file 3: Fig. S3. K222suc reduces the degradation of LDHA in HGC27 cells. a Flag-K222E (K222suc mimic) fusion protein is stably expressed in HGC27 cells after transfection. b-d Flag-K222E protein was accumulated in HGC27 cells and K222suc could inhibit the lysosomal degradation of LDHA. After transfection with Flag-K222E plasmid for 24 h, HGC27 cells were treated with CHX (10 μg/ml) (b), MG132 (10 mM) (c), or BafA1 (20 nM) (d). The cell lysates were collected at the indicated time points, and the K222E fusion protein was detected using anti-Flag antibody. Data are presented as mean ± SEM, *P < 0.05, **P < 0.01, ***P < 0.001. [file 13046_2020_1681_MOESM3_ESM.tif]
