# Supplementary material for: Lysine-222 succinylation reduces lysosomal degradation of lactate dehydrogenase a and is increased in gastric cancer
Source: J Exp Clin Cancer Res. 2020 Aug 28;39:172. doi: 10.1186/s13046-020-01681-0 (PMC7455916; doi:10.1186/s13046-020-01681-0)
Supplement: Supplementary file 4 — Additional file 4: Table S1. PTM sites in human LDHA (PhosphoSitePlus). [file 13046_2020_1681_MOESM4_ESM.docx]

**Table S1 PTM sites in human LDHA (PhosphoSitePlus)**

| PTM site | Sequence |
| --- | --- |
| T3-p | MAtLkDQLIy |
| K5-ac | MAtLkDQLIyNL |
| K5-m1 | MAtLkDQLIyNL |
| K5-ub | MAtLkDQLIyNL |
| K5-sc | MAtLkDQLIyNL |
| Y10-p | tLkDQLIyNLLkEEQ |
| K14-ac | QLIyNLLkEEQtPQN |
| K14-ub | QLIyNLLkEEQtPQN |
| K14-sm | QLIyNLLkEEQtPQN |
| K14-sc | QLIyNLLkEEQtPQN |
| T18-p | NLLkEEQtPQNkITV |
| K22-ac | EEQtPQNkITVVGVG |
| K57-ac | LVDVIEDkLkGEMMD |
| K57-ub | LVDVIEDkLkGEMMD |
| K59-ub | DVIEDkLkGEMMDLQ |
| T74-p | HGSLFLRtPkIVSGk |
| K76-ub | SLFLRtPkIVSGkDy |
| K76-sc | SLFLRtPkIVSGkDy |
| K81-ac | tPkIVSGkDyNVTAN |
| K81-ub | tPkIVSGkDyNVTAN |
| K81-sc | tPkIVSGkDyNVTAN |
| Y83-p | kIVSGkDyNVTANsk |
| S89-p | DyNVTANskLVIITA |
| K90-ub | yNVTANskLVIITAG |
| K118-ac | QRNVNIFkFIIPNVV |
| K118-m1 | QRNVNIFkFIIPNVV |
| K118-ub | QRNVNIFkFIIPNVV |
| K118-sc | QRNVNIFkFIIPNVV |
| K126-ac | FIIPNVVkysPNCkL |
| K126-ub | FIIPNVVkysPNCkL |
| K126-sc | FIIPNVVkysPNCkL |
| Y127-p | IIPNVVkysPNCkLL |
| S128-p | IPNVVkysPNCkLLI |
| K132-ac | VkysPNCkLLIVSNP |
| K132-ub | VkysPNCkLLIVSNP |
| Y145-p | NPVDILTyVAWKISG |
| K155-ub | WKISGFPkNRVIGsG |
| S161-p | PkNRVIGsGCNLDSA |
| Y172-p | LDSARFRyLMGERLG |
| S184-p | RLGVHPLsCHGWVLG |
| K212-ub | NVAGVSLkTLHPDLG |
| K222-ac | HPDLGTDkDkEQWkE |
| K222-ub | HPDLGTDkDkEQWkE |
| K222-sc | HPDLGTDkDkEQWkE |
| K224-ac | DLGTDkDkEQWkEVH |
| K224-ub | DLGTDkDkEQWkEVH |
| K228-ac | DkDkEQWkEVHkQVV |
| K228-ub | DkDkEQWkEVHkQVV |
| K228-sc | DkDkEQWkEVHkQVV |
| K232-ac | EQWkEVHkQVVEsAy |
| K232-ub | EQWkEVHkQVVEsAy |
| K232-sc | EQWkEVHkQVVEsAy |
| S237-p | VHkQVVEsAyEVIkL |
| Y239-p | kQVVEsAyEVIkLkG |
| K243-ac | EsAyEVIkLkGYtsW |
| K243-ub | EsAyEVIkLkGYtsW |
| K245-ub | AyEVIkLkGYtsWAI |
| T248-p | VIkLkGYtsWAIGLS |
| S249-p | IkLkGYtsWAIGLSV |
| R269-m1 | SIMKNLRrVHPVSTM |
| K278-ac | HPVSTMIkGLYGIkD |
| K278-m1 | HPVSTMIkGLYGIkD |
| K278-ub | HPVSTMIkGLYGIkD |
| K278-sc | HPVSTMIkGLYGIkD |
| K284-ub | IkGLYGIkDDVFLSV |
| K284-sc | IkGLYGIkDDVFLSV |
| K305-ub | NGISDLVkVTLtsEE |
| T309-p | DLVkVTLtsEEEARL |
| S310-p | LVkVTLtsEEEARLk |
| K317-ub | sEEEARLkksADtLW |
| K318-ac | EEEARLkksADtLWG |
| K318-ub | EEEARLkksADtLWG |
| S319-p | EEARLkksADtLWGI |
| T322-p | RLkksADtLWGIQkE |
| K328-ac | DtLWGIQkELQF |
| K328-ub | DtLWGIQkELQF |
| K328-sm | DtLWGIQkELQF |
| K328-sc | DtLWGIQkELQF |

ac: acetylation, m1: mono-methylation, p: phosphorylation, sc: succinylation, sm: sumoylation, ub: ubiquitylation.

(<https://www.phosphosite.org/siteTableNewAction?id=4103&showAllSites=true>)
